# Effect of ABCG2/BCRP Expression on Efflux and Uptake of Gefitinib in NSCLC Cell Lines

**DOI:** 10.1371/journal.pone.0141795

**Published:** 2015-11-04

**Authors:** Maricla Galetti, Pier Giorgio Petronini, Claudia Fumarola, Daniele Cretella, Silvia La Monica, Mara Bonelli, Andrea Cavazzoni, Francesca Saccani, Cristina Caffarra, Roberta Andreoli, Antonio Mutti, Marcello Tiseo, Andrea Ardizzoni, Roberta R. Alfieri

**Affiliations:** 1 Department of Clinical and Experimental Medicine, University of Parma, Parma, Italy; 2 Italian Workers’ Compensation Authority (INAIL), Research Centre at the University of Parma, Parma, Italy; 3 Division of Medical Oncology, University Hospital of Parma, Parma, Italy; 4 Medical Oncology Unit, S. Orsola-Malpighi Hospital, Bologna, Italy; Roswell Park Cancer Institute, UNITED STATES

## Abstract

**Background:**

BCRP/ABCG2 emerged as an important multidrug resistance protein, because it confers resistance to several classes of cancer chemotherapeutic agents and to a number of novel molecularly-targeted therapeutics such as tyrosine kinase inhibitors. Gefitinib is an orally active, selective EGFR tyrosine kinase inhibitor used in the treatment of patients with advanced non small cell lung cancer (NSCLC) carrying activating EGFR mutations. Membrane transporters may affect the distribution and accumulation of gefitinib in tumour cells; in particular a reduced intracellular level of the drug may result from poor uptake, enhanced efflux or increased metabolism.

**Aim:**

The present study, performed in a panel of NSCLC cell lines expressing different ABCG2 plasma membrane levels, was designed to investigate the effect of the efflux transporter ABCG2 on intracellular gefitinib accumulation, by dissecting the contribution of uptake and efflux processes.

**Methods and Results:**

Our findings indicate that gefitinib, in lung cancer cells, inhibits ABCG2 activity, as previously reported. In addition, we suggest that ABCG2 silencing or overexpression affects intracellular gefitinib content by modulating the uptake rather than the efflux. Similarly, overexpression of ABCG2 affected the expression of a number of drug transporters, altering the functional activities of nutrient and drug transport systems, in particular inhibiting MPP, glucose and glutamine uptake.

**Conclusions:**

Therefore, we conclude that gefitinib is an inhibitor but not a substrate for ABCG2 and that ABCG2 overexpression may modulate the expression and activity of other transporters involved in the uptake of different substrates into the cells.

## Introduction

ATP-binding cassette (ABC) transporters, such as P-glycoprotein/multidrug resistance 1/ABCB1 (P-gp/MDR1/ABCB1) and breast cancer resistance protein (BCRP, also known as ABCG2), are membrane proteins that pump out of the cells a variety of structurally unrelated substrates in an energy-dependent manner [1]. ABCG2 is a half-molecule ABC transporter with an NH_2_-terminal ATP binding site and a COOH-terminal transmembrane domain [2, 3], which may act as a homodimer [4] or homotetramer [5]. ABCG2 is expressed in various tissues involved in adsorption, distribution, and elimination of drugs and metabolites [6]. In addition, ABCG2 is overexpressed in several cell lines selected in the presence of anticancer drugs and functions as a key player in the multidrug-resistance phenotype of cancer cells [7].

ABCG2 has a potent ability to interact with numerous clinically important tyrosin kinase inhibitors (TKIs) including imatinib, nilotinib, dasatinib, lapatinib, sunitinib, canertinib, erlotinib and gefitinib [8–14]. Many TKIs are ABC transporter substrates at low concentrations while they are inhibitors at higher concentrations, so the same compound may act both as a substrate or an inhibitor depending on its concentration [15].

Gefitinib is an orally active, selective epidermal growth factor receptor (EGFR) tyrosine kinase inhibitor (TKI) used in the treatment of patients with advanced NCSLC. Tumors having EGFR activating mutations are associated with an enhanced response, however, acquired resistance occurs in virtually all NSCLC tumors that initially respond to gefitinib therapy [16–18].

The interaction of gefitinib with the efflux transporter ABCG2 has been studied by several groups in the last years, leading to conflicting results. Some studies have reported gefitinib as a substrate actively extruded by ABCG2 [19, 20]. In addition high expression of ABCG2 has been shown to confer acquired resistance to gefitinib and it has been correlated with the efflux of gefitinib from the cells [21]. In contrast, Steward C. et al. [22] found that gefitinib is a potent inhibitor but not a substrate of ABCG2. Moreover, gefitinib has been demonstrated to reverse ABCG2-mediated multidrug resistance in preclinical models [23, 24] and the underlying mechanism has been related to a direct inhibition of the transporter [22, 25, 26]. Collectively these studies suggest that gefitinib is actually a potent inhibitor of ABCG2, but the role of ABCG2 in gefitinib efflux still remains controversial.

Most of the studies on ABCG2-drug interaction have been performed in ABCG2 overexpressing cell models. These studies, however, do not take into account that a forced expression of efflux proteins may affect the expression and activity of endogenous transporters, as recently reported. In particular, the overexpression of efflux proteins (MDR1, MRP2 and ABCG2) was shown to alter the gene and protein expression as well as the functional activity of the endogenous influx peptide transporter system (PepT) in MDCK cells. The influx of Gly-Sar, the tipical substrate for peptide transporter, and the level of mRNA for PepT1 and 2 were significantly reduced in overexpressing cells in comparison with parental cells [27].

In view of our previous works on gefitinib uptake [28] and metabolism [29] in NSCLC cell lines, and considering our experience on aminoacid [30] and nutrient transport [31], in this paper we characterized the efflux of gefitinib in a panel of NSCLC cell lines, we analyzed the effect of ABCG2 silencing on accumulation, efflux and uptake of gefitinib and the effect of ABCG2 overexpression on the regulation of a number of drug transporter genes and on the uptake of gefitinib and of various metabolites.

Our present findings further indicate that gefitinib is an inhibitor but not a substrate of ABCG2 and might provide potentially important information regarding the effect of ABCG2 overexpression on the expression and activity of other transporters involved in the uptake of different substrates into the cells.

## Materials and Methods

### Cell culture

The human NSCLC cell lines HCC827, H292, H460, H1299 A549 and Calu-1 were cultured in RPMI 1640; SKMES-1 and SKLU-1 were cultured in MEM with 1mmol/L sodium pyruvate and 0.1mmol/L nonessential amino acids. All media were supplemented with 2 mM glutamine, 10% fetal bovine serum (FBS Gibco, Life Technologies). Cell lines were from American Type Culture Collection (Manassas, VA, USA) and were maintained under standard cell culture conditions at 37°C in a water-saturated atmosphere of 5% CO_2_ in air. The HCC827 cell line was kindly provided by Dr P. Janne (Dana-Farber Cancer Institute, Boston MA). HEK293 cells stably transfected with ABCG2 [32] were kindly provided by Dr. Maurizio Viale (IRCCS AOU S. Martino-IST, Genoa, Italy) and were cultured in DMEM supplemented with G418 sulfate (2 mg/mL, Sigma Aldrich, St. Louis, MO, USA).

### Drug treatment

Gefitinib (ZD1839/Iressa^®^) was provided by AstraZeneca (Milan, Italy). In all assays, the drug was dissolved in DMSO immediately before its addition to the media. The concentration of DMSO never exceeded 0.1% (v/v) and equal amounts of the solvent were added to control cells. The ABCG2 inhibitors Fumitremorgin C and Ko143 were purchased from Sigma Aldrich (St. Louis, MO, USA), while the MDR1 inhibitor PSC833 was kindly provided by Novartis.

### Western immunoblot analysis

Cell protein extraction, solubilization, and analysis by 1-D PAGE were performed as previously described [33]. The antibody against ABCG2 clone BXP-21 (diluition 1:1000) was from Abcam (Cambridge, UK). The antibody against GAPDH was from Ambion (Austin, TX). HRP-conjugated secondary antibodies were from Pierce (Rockford, IL) and the chemiluminescence system (ImmobilionTM Western Cemiluminescent HRP Substrate), was from Millipore (Temecula, CA).

### Flow cytometry

One million of cells were incubated with Isotype control Monoclonal Mouse IgG2/R-PE clone MPC-11, 5μl/1x10^6^ cells (Ancell IRP, Bayport, MN, USA) or PE mouse anti-Human ABCG2 clone 5D3, 10μl/1x10^6^ cells (BD Biosciences, San Josè CA) to determine ABCG2 protein membrane levels, as previously described [34]. After one hour of incubation the analysis was performed using a Beckman-Coulter EPICS-XL flow cytometer. Mean fluorescence intensity (MFI) values were converted in units of equivalent fluorochrome (MEF) using the FluoroSpheres 6-Peak Kit and following the manufacturer’s recommended procedure (Dako, CA, USA). This method makes it possible to transform directly the relative channel number obtained by flow cytometry analysis into the number of molecules of equivalent soluble fluorochromes by eliminating variation due to cytometer setting, day to day performance variability and differences in stimulation dependent autofluorescence levels. We used a FluoroSpheres kit (DAKO).

### Hoechst 33342 accumulation and efflux

To evaluate the functionality of ABCG2 protein, we used the Hoechst 33342 dye accumulation assay. Hoechst 33342 is a cell-permeable hydrophobic dye, which is a characterized substrate actively extruded by ABCG2 and becomes fluorescent only in a complex with DNA [35].

Cells were plated at a density of 10,000 cells/well in 96-well plates (Perkin Elmer, Boston, USA) and incubated for 48 hours in complete growth medium at 37°C. After removing the medium, cells were incubated in phenol red-free medium with 1 μM Hoechst 33342 substrate for 4 hours at 37°C, in the presence or absence of the inhibitors Fumetrimorgin C or Ko143. Then cells were washed twice with ice-cold PBS, and Hoechst 33342 dye accumulation was measured in a fluorescence spectrophotometer (EnSpire Multimode Plate Readers, Perkin Elmer, Boston, USA) at 350nm (excitation)/460nm (emission). Trichloroacetic acid (TCA, 5%, w/v) was then added to precipitate cell proteins, which were further dissolved in 0.5 N NaOH and their concentration was determined by a dye-fixation method (Bio-Rad) using bovine serum albumin as a standard [36]. The results were expressed as absorbance at 350nm/460nm, per microgram of protein.

For efflux study, cells were incubated with 1 μM Hoechst 33342 substrate for 4 hours at 37°C, followed by a subsequent substrate-free efflux period in the presence or absence of the inhibitors. Hoechst 33342 efflux was expressed as percentage of the value determined after 4 hours of incubation.

Relative ABCG2 activity was defined as the ratio of Hoechst 33342 accumulation per μg of protein between Fumetrimorgin C or Ko143 treated and untreated cells and was expressed as fold increase.

### Gefitinib accumulation and uptake

Labeled [^3^H]gefitinib (7.64 Ci/mmol) was custom made by Perkin Elmer (Boston, MA, USA). In all the experiments the radiolabeled drug was mixed with unlabeled drug to reach the desired final concentration. The measurements of [^3^H]gefitinib accumulation by cells was essentially performed as described previously for amino acids [30] and creatine [31], with minor modifications. Cells were seeded into 4 cm^2^ wells of disposable multiwell trays (NUNC, Rosekilde, Danmark) to give the desired cell density, and incubated for 48 hours in complete growth medium at 37°C. After removing the medium, the cells were quickly washed with RPMI containing 5% fetal bovine serum, 2 mM glutamine, 10 mM HEPES buffer (accumulation buffer) and immediately incubated in accumulation buffer at 37°C for the desired period of time, in the presence of labelled gefitinib and ABC inhibitors.

Initial velocity (5min) of [^3^H]gefitinib uptake was measured in accumulation buffer, in the presence or absence of the ABC inhibitors.

The incubations were stopped by removal of the accumulation buffer and the cells were quickly washed three times with cold Earle’s balanced salt solution containing 0.1% glucose. TCA (5%, w/v) was added to denature the cells and the radioactivity in samples of the acid extracts was measured by scintillation counting. Cell proteins, precipitated by TCA, were dissolved in 0.5N NaOH and their concentration determined by a dye-fixation method (Bio-Rad) using bovine serum albumin as a standard.

### Glutamine, Glucose and MPP uptake

Labeled N-[Methyl-3H]MPP(+) (86.4 Ci/mmol), L-[3,4-3H(N)]Glutamine (46.1 Ci/mmol) and 2[1,2-3H (N)]Deoxy-D-glucose (20 Ci/mmol) were purchased from Perkin Elmer (Boston, MA, USA). After removing the culture medium, the cells were quickly washed with Earle’s balanced salt solution containing 0.1% glucose (uptake buffer) and immediately incubated in uptake buffer at 37°C for 5 min in the presence of radiolabelled compounds (1μCi/ml for N-[Methyl-3H]MPP(+), L-[3,4-3H(N)]Glutamine and 2[1,2-3H (N)]Deoxy-D-glucose. The incubations were stopped by removal of the uptake buffer and the cells were quickly washed three times with fresh cold solution. The cellular radiolabel content was determined in the TCA soluble fraction by scintillation counting. Cell proteins, precipitated by TCA, were dissolved in 0.5N NaOH and their concentration determined by a dye-fixation method (Bio-Rad) using bovine serum albumin as a standard.

### Gefitinib efflux

Cells were incubated with [^3^H]gefitinib for 4 hours, in the presence or absence of ABC inhibitors and then the medium was replaced with drug-free medium to determine the percentage of drug efflux. The inhibitors were maintained also during the efflux time. Then cells were quickly washed three times with cold Earle’s balanced salt solution containing 0.1% glucose. The cellular radiolabel content was determined in the TCA soluble fraction by scintillation counting. Cell proteins, precipitated by TCA, were dissolved in 0.5N NaOH and their concentration determined by a dye-fixation method (Bio-Rad) using bovine serum albumin as a standard. gefitinib efflux was expressed as percentage of the value determined after 4hours of gefitinib incubation.

### RNA interference assay

Cells were transfected with Invitrogen Stealth^™^ siRNA (Invitrogen, Carlsbad, CA) against ABCG2 (1:1:1 mixture of ^#^HSS114013, ^#^HSS114014 and ^#^HSS114015 or ^#^HSS114013 alone) or negative control with a final concentration of 90nM. The transfection was carried out according to the Invitrogen forward transfection protocol for Lipofectamine^™^ RNAiMAX transfection reagent. After 48 hours of transfection, the medium was aspirated and replaced with the appropriate medium for subsequent analyses.

### Liquid chromatography tandem mass spectrometry (LC-MS/MS)

LC-MS/MS analysis was performed as previously described [29]. Briefly, gefitinib was extracted from cells with absolute EtOH at 4°C, cell extracts were centrifuged (4°C, 10,000 g, 5 min) and collected. A fixed volume of ethanolic extract was diluted with five volume of internal standard in aqueous formic solution and injected into the HPLC-MS/MS system. Cell proteins were quantified after solubilization in NaOH 0.5 N by the Bradford method.

LC analyses were carried out with an Agilent HP 1100 pump coupled with a API4000 triple-quadrupole mass spectrometer (AB SCIEX, Framingham, MA, USA) equipped with a TurboIonSpray^™^ interface operating in Selected Reaction Monitoring (SRM) mode. Chromatography was performed on a Synergi Hydro-RP column (5 x 2.0mm i.d., 2μm; Phenomenex) using variable proportions of 5mM aqueous formic acid and methanol/acetonitrile (95/5, v/v) mixture as the mobile phase.

The analytes were ionized in positive ion mode and the following SRM transitions were monitored: m/z 447 ([M+H]^+^) → 128 for gefitinib; and m/z 394 ([M+H]^+^) → 336 for Internal Standard. erlotinib was used as Internal Standard.

To validate the analytical method all the experiments were done in matrix (ethanolic extract from untreated cells). Working calibrations were performed in matrix and calibrating standards, obtained adding gefitinib solutions at five different levels, were treated as samples. Calibration curves were built by linear regression analysis of the area ratios analyte/IS versus the concentration of analytes injected. Linearity of gefitinib MS response was observed over 3-4 orders. The limit of detection, defined as signal to noise ratios equal to three (S/N = 3) was 2 nM. The imprecision of the method, calculated as %RSD at three different concentration levels (n = 6) in matrix, was within 3–6% for intra-day and 4–9% for inter-day determinations.

### RT^2^ profiler PCR array of human drug transporters

Quantitative expression of various ABC and SLC transporters was determined using the RT^2^ Profiler human drug transporter PCR Arrays manufactured by Qiagen/SA Biosciences (Cat n. 330231 PAHS-070).

RNA was isolated using RNeasy spin columns using RNeasy Minikit (Qiagen). cDNA was synthesized from 0.5 μg of RNA using RT^2^ PCR Array First Strand Kit (Qiagen/SA Biosciences) and the arrays were performed according to the manufacturer instructions using Qiagen/SA Biosciences Master Mix. The experiment was repeated twice.

The arrays included 84 transporter genes, 5 housekeeping genes, reverse transcription efficiency and DNA contamination controls.

For each gene, ΔCt was calculated using the same threshold (0.2) for all genes and Ct>35 considered as no expression, followed by normalization to 5 housekeeping genes (Actin beta, beta-2-microglobulin, GAPDH, HPRT1 and RPLP0) included in each array. The fold change was calculated by the ΔΔC_T_ method using the program provided by Qiagen/SA Biosciences.

### Statistical Analysis

Statistical analyses were carried out using GraphPad Prism version 5.00 software (GraphPad Software Inc., San Diego, CA). Results are expressed as mean values ± standard deviations (SD) for the indicated number of independent measurements.

The significance of differences between the mean values recorded for different experimental conditions was calculated by the Student's t-test and comparison among groups was made using analysis of variance (one-way ANOVA repeated measures) followed by Bonferroni’s post-test. P values are indicated where appropriate in the figures and in their legends. A P-value < 0.05 was considered as significant.

## Results

### Characterization of ABCG2 expression and activity in NSCLC cell lines

**Fig 1:**
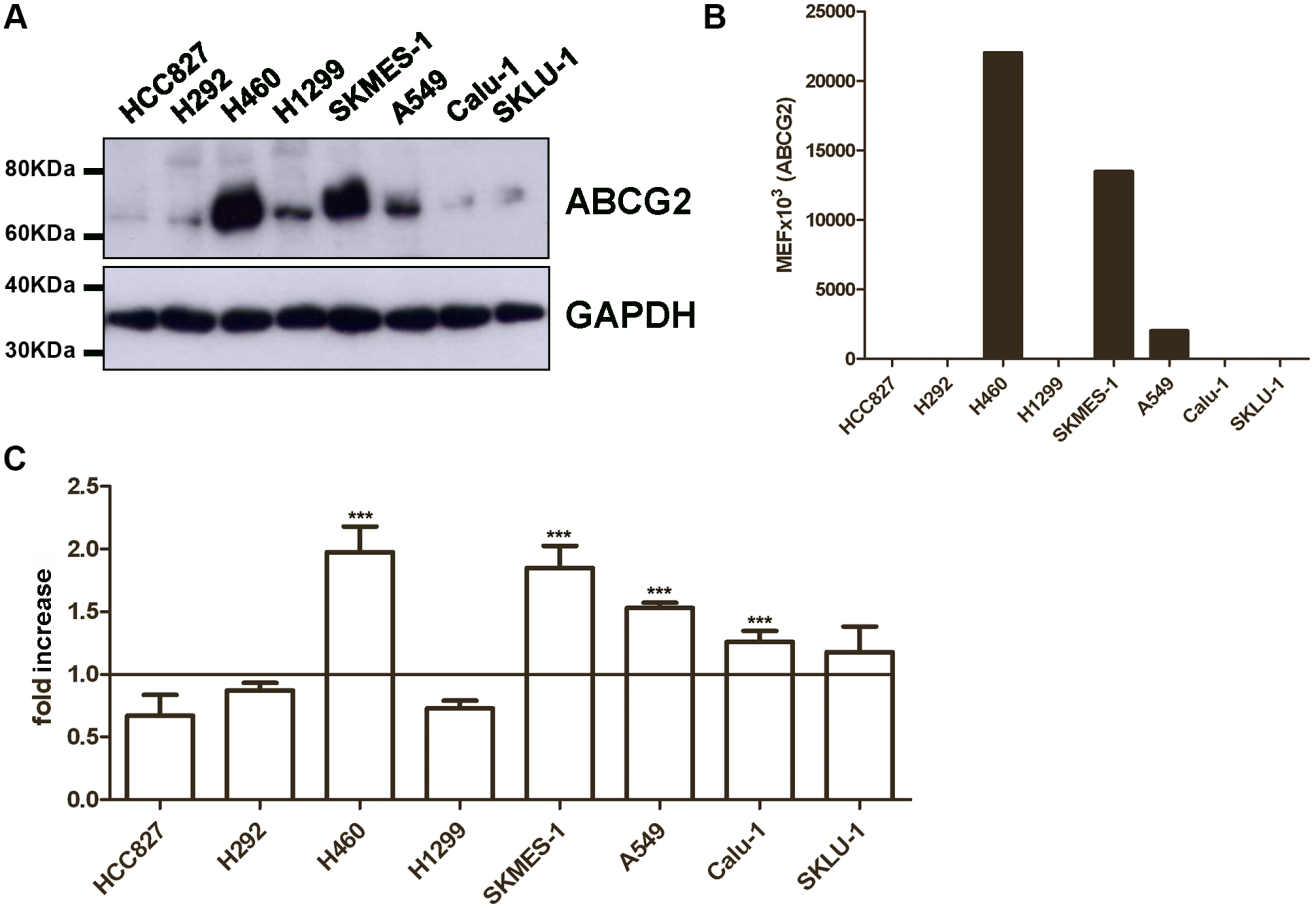
Characterization of ABCG2 expression and activity in NSCLC cell lines. (A) Cells were lysed and 50μg of proteins loaded to assess ABCG2 protein expression by western blot analysis. Data are from a representative experiment. Each experiment, repeated three times, yielded similar results. (B) ABCG2 protein levels on cell surface were quantified by flow-cytometry and expressed as molecular equivalent of fluorochrome (MEF) as described in the Methods Section. (C) Cells were loaded with 1 μM Hoechst 33342 in the presence or in the absence of 10 μM Fumitremorgin C. After 4 hours of incubation, Hoechst 33342 was removed and its fluorescence was determined by luminometer. Relative ABCG2 activity was defined as the ratio of Hoechst 33342 accumulation per μg of protein between Fumetrimorgin C treated cells and untreated cells and was expressed as fold increase. The mean values of three independent measurements (± SD) are shown (***P < 0.001).

We first evaluated ABCG2 expression as total protein level (Fig 1A) and plasma membrane level (Fig 1B) in a panel of NSCLC cell lines showing sensitivity (HCC827, H292) or resistance (H460, H1299, SKMES-1, A549, Calu-1 and SKLU-1) to gefitinib. ABCG2 expression, evaluated by Western blotting and flow cytometry, varied widely among the analyzed cell lines, with H460 and SKMES-1 cells expressing the highest levels.

To assess the activity of ABCG2 protein, we measured the accumulation of Hoechst 33342, a known ABCG2 substrate, in the absence or presence of the specific ABCG2 inhibitor, Fumitremorgin C (Fig 1C). After 4 hours of incubation with 1 μM Hoechst 33342 in the presence of 10 μM Fumitremorgin C, H460, SKMES-1, and A549 cell lines showed a 1.5-2-fold increase in the intracellular dye level, confirming the ABCG2 functionality in these cell lines.

### Effect of gefitinib on ABCG2 activity and expression

Since it has been previously reported that gefitinib reversed ABCG2-mediated anticancer drug resistance by inhibiting the transporter function of ABCG2 in cells overexpressing ABCG2 [25], we investigated whether gefitinib inhibited the transporter function of ABCG2 also in non-drug selected cell lines.

**Fig 2:**
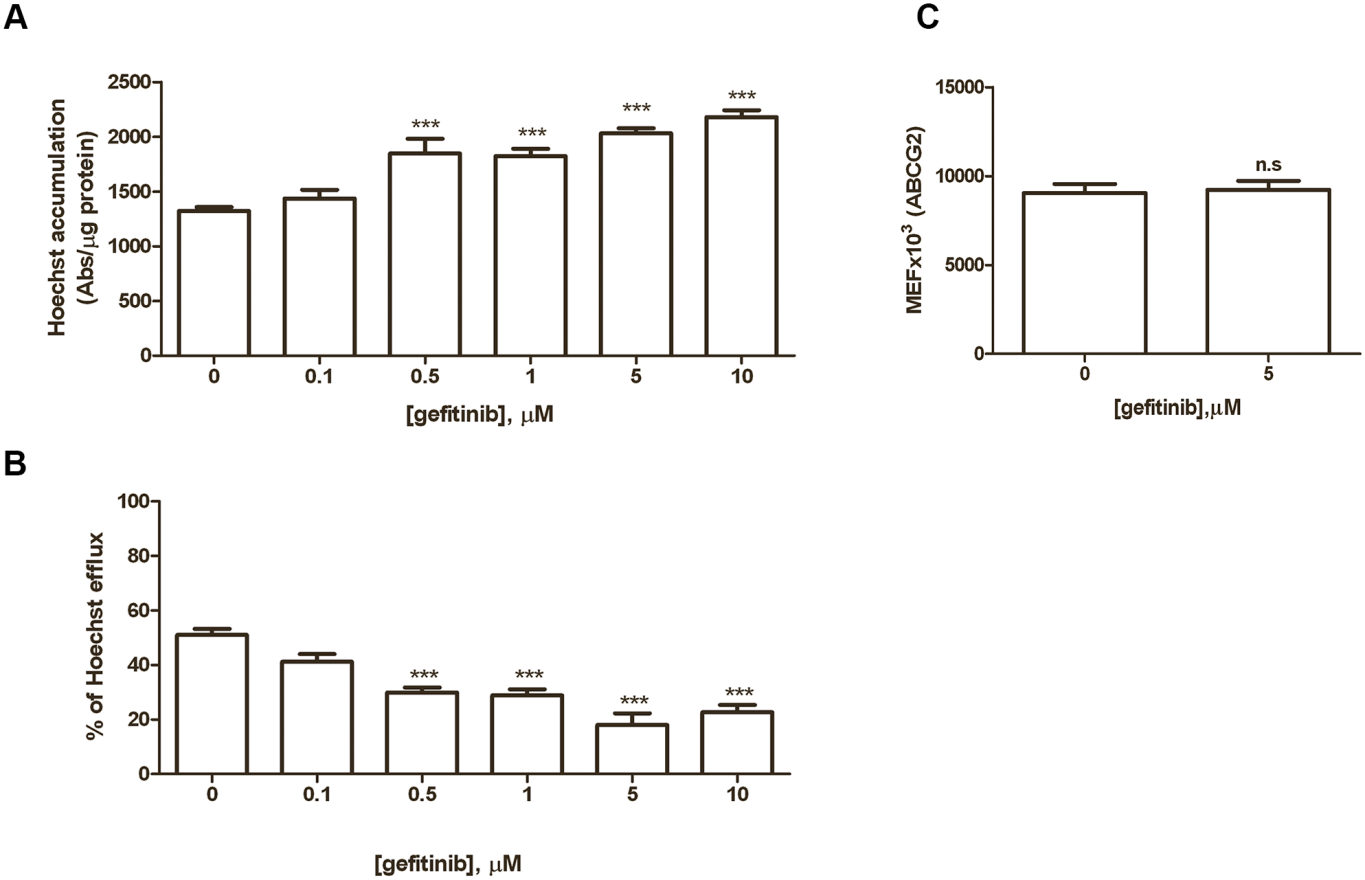
Effect of gefitinib on ABCG2 activity and protein expression. H460 cells were loaded with 1 μM Hoechst 33342 in the presence of the indicated concentrations of gefitinib. (A) After 4 hours, Hoechst 33342 was removed and fluorescent dye accumulation was expressed as absorbance per μg of protein. (B) The percentage of efflux was calculated after a further incubation of 1 hour in phenol red-free medium in the presence of the indicated concentration of gefitinib. Values are the means (± SD) of three independent determinations; one-way ANOVA followed by Bonferroni post-test (*** P < 0.001) (C) H460 cells were treated with 5 μM gefitinib for 4 hours; then cell surface expression of ABCG2 was evaluated by flow cytometry and the quantification is reported as molecular equivalent of fluorochrome (MEF). The mean values of three independent measurements (± SD) are shown.

The intracellular Hoechst 33342 accumulation was evaluated in H460 cells after 4 hours of incubation with increasing concentrations of gefitinib (Fig 2A). Intracellular Hoechst 33342-derived fluorescence was increased in cells treated with gefitinib in a dose-dependent manner starting from 0.5 μM; correspondingly the percentage of Hoechst 33342 efflux decreased (Fig 2B), indicating that gefitinib, interacting with ABCG2, inhibited its transporter function. This inhibition was not related to a reduction in the ABGC2 level in the plasma membrane at least at 5 μM (Fig 2C).

### Effects of different ABGC2 and MDR1 inhibitors on intracellular gefitinib content and efflux

**Fig 3:**
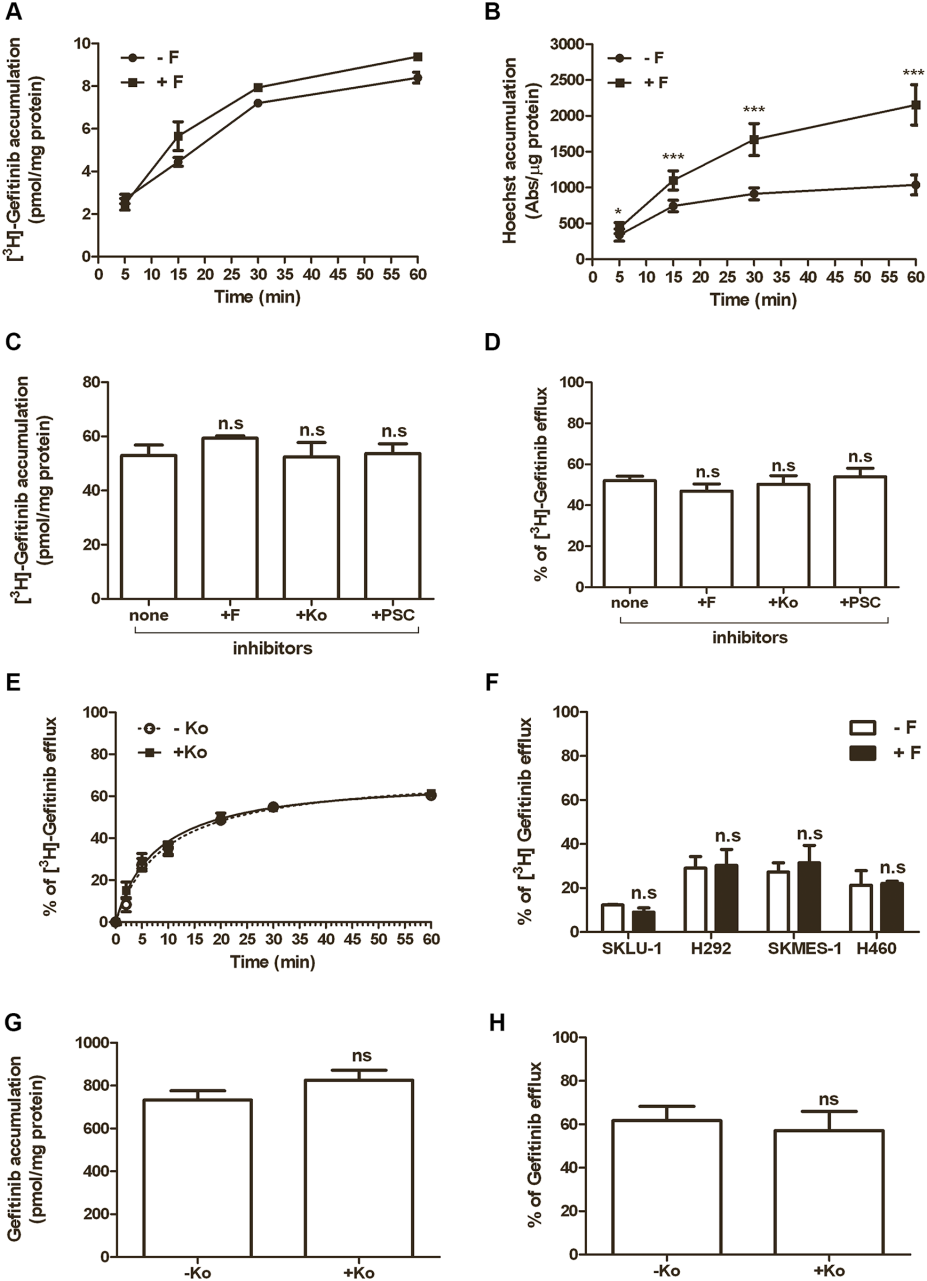
Effects of different ABGC2 and MDR1 inhibitors on gefitinib accumulation and efflux. Time course of 10 nM [^3^H]gefitinib (A) and 1 μM Hoechst 33342 (B) accumulation in H460 cells, incubated in the presence or absence of 10 μM Fumitremorgin C. [^3^H]gefitinib was expressed as pmol/mg of protein and Hoechst 33342 accumulation was expressed as absorbance per μg of protein. Each point represents the mean (± SD) of four independent determinations (* P<0.05; ***P < 0.001). (C) H460 cells were incubated with 0.1 μM [^3^H]gefitinib for 4 hours in the absence or in the presence of 10 μM Fumitremorgin C (F) or 1 μM Ko143 (Ko) (ABCG2 inhibitors), or 10 μM PSC833 (PSC) (MDR1 inhibitor). At the end of the treatment, [^3^H]gefitinib accumulation was measured. After 4 hours of incubation in the presence of 0.1 μM [^3^H]gefitinib, the cells were washed, incubated in drug-free culture medium with or without the inhibitors and the percentage of efflux was calculated after 1 hour of incubation (D) or at the indicated times (E). (F) SKLU-1, H292, SKMES-1and H460 were incubated with 0.1 μM [^3^H]gefitinib for 4 hours, in the presence or absence of 10 μM Fumitremorgin C. The percentage of efflux was calculated after a further incubation of 5min in gefitinib-free normal growth medium, in the presence or in the absence of the inhibitor. (G-H) Intracellular level of gefitinib in H460 cell line was evaluated by LC-MS/MS, in the same condition described in panels C-D. Values given are the means (± SD) of three independent determinations; one-way ANOVA followed by Bonferroni post-test.

In order to evaluate whether the inhibition of ABCG2 activity by specific inhibitors was associated with changes in the intracellular gefitinib levels, we analyzed the time course of [^3^H]gefitinib accumulation at a very low concentration (10nM) in H460 cells, in the absence or presence of 10 μM Fumitremorgin C. Fig 3A shows a progressive increase of the level of intracellular gefitinib. However, the ABCG2 inhibitor did not significantly change gefitinib accumulation, despite it effectively inhibited the ABCG2 transporter, as demonstrated by the rapid increase of intracellular Hoechst 33342 levels, that were significantly higher than in untreated cells (Fig 3B). Taken together these results suggest that in cultured NSCLC cells, inhibition of ABCG2 is associated with increased intracellular levels of the substrate Hoechst 33342, but not with an altered accumulation of intracellular radiolabeled gefitinib.

In an attempt to better characterize the role of ABC transporters on both accumulation and efflux of gefitinib, we used various inhibitors of these transporters. Firstly, we determined the intracellular levels of [^3^H]gefitinib after dosing H460 cells with the ABCG2 inhibitors Fumitremorgin C and Ko143 or the MDR1 inhibitor PSC833. As reported in Fig 3C, neither Fumitremorgin C nor Ko143 significantly affected the gefitinib content after 4 hours of treatment. Moreover, even the inhibition of the MDR1 pump by PSC833 was ineffective in enhancing the intracellular level of gefitinib. Then we analyzed the effect of these drugs on gefitinib efflux. After 4 hour of [^3^H]gefitinib treatment in the absence/presence of ABC-inhibitors, the external [^3^H]gefitinib was removed and the cells were maintained in gefitinib-free growth medium for further 1 hour, in the absence/presence of the inhibitors. As shown in Fig 3D, control cells showed a decrease in the level of intracellular gefitinib by around 50%. Treatment with Fumitremorgin C, Ko143 or PSC833 did not change the percentage of efflux of the radiolabeled gefitinib as compared with control cells and the efflux was time dependent with a plateau starting from 20 min (Fig 3E).

Interestingly, as shown in Fig 3F, no significant modulation of gefitinib efflux was observed in the absence or presence of Fumitremorgin C in various NSCLC cell models expressing different ABCG2 levels (see Fig 1A and 1B).

The results that ABCG2 inhibition has no effect on both intracellular gefitinib accumulation and efflux in H460 cells were also confirmed by LC-MS/MS analysis (Fig 3G and 3H).

### Effect of ABCG2 silencing or overexpression on gefitinib accumulation, efflux and uptake

**Fig 4:**
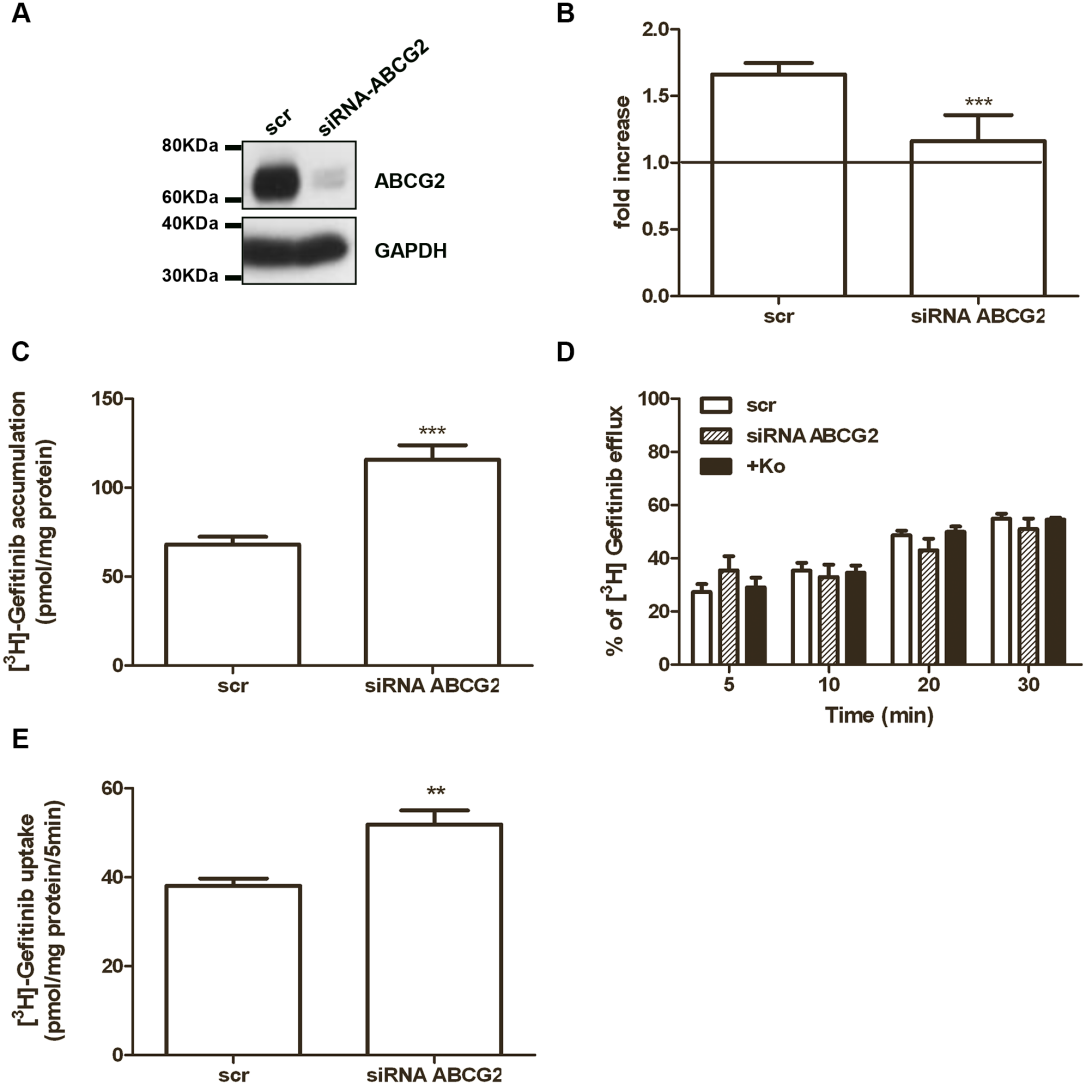
Effect of ABCG2 silencing on [^3^H]gefitinib accumulation, efflux, and uptake. H460 cells were transfected with ABCG2 siRNA (1:1:1 mixture of ^#^HSS114013, ^#^HSS114014 and ^#^HSS114015) or control siRNA (scr) for 48 hours and then analyzed for ABCG2 expression by Western blotting (A) or ABCG2 activity (B). Cells were incubated for 4 hours with 1 μM Hoechst 33342 in the presence or in the absence of Fumetrimorgin C and the relative ABCG2 activity was calculated as the ratio of Hoechst 33342 accumulation per μg of protein between Fumitremorgin C treated cells and untreated cells, and expressed as fold increase. Data are expressed as mean (± SD) of three different experiments (***P < 0.001). In H460 transfected cells, radiolabeled gefitinib accumulation was measured after 4 hours of treatment with 0.1 μM [^3^H]gefitinib (C), then the medium was replaced with fresh medium and the percentage of efflux was calculated after 5, 10, 20 and 30 min in the presence or absence of 1 μM Ko143 (***P < 0.001) (D). Initial velocity (5 min) of [^3^H] gefitinib uptake was measured after 4 hours of 0.1 μM gefitinib treatment (E). Each bar represents the mean (± SD) of four independent determinations. (** P < 0.01).

We analyzed the effect of ABCG2 silencing on [^3^H]gefitinib accumulation and efflux in H460 cells. ABCG2 silencing, using RNA interference, significantly inhibited both ABCG2 expression (Fig 4A) and activity (Fig 4B). As reported in Fig 4C, we found that knock-down of ABCG2 resulted in an increase of intracellular [^3^H]gefitinib levels compared to the negative siRNA-scramble control. This result was in contrast with the data obtained by using the inhibitors shown in Fig 3C; however, when we analyzed the percentage of efflux during time, we observed that gefitinib efflux was not affected by ABCG2 silencing or by the presence of 1 μM Ko143 (Fig 4D).

Interestingly, when we silenced ABCG2 expression, the initial velocity (5 min) of [^3^H]gefitinib uptake was significantly increased (Fig 4E), suggesting that ABCG2 expression is involved in controlling [^3^H]gefitinib uptake rather than in [^3^H]gefitinib efflux.

To rule out that the results were related to off-target effects due to the use of a pool of three siRNAs, we performed additional functional experiments with a single siRNA (^#^HSS114013). As shown in S1 Fig, this single siRNA reduced the ABCG2 expression and activity with subsequent increase of intracellular [^3^H]gefitinib levels and uptake, confirming the results obtained with siRNA mix.

To further validate the relevance of ABCG2 expression in [^3^H]gefitinib uptake, we used HEK293/R2 cells over-expressing the ABCG2 transporter, obtained by stably transfecting HEK293 cells with an ABCG2 expression vector.

**Fig 5:**
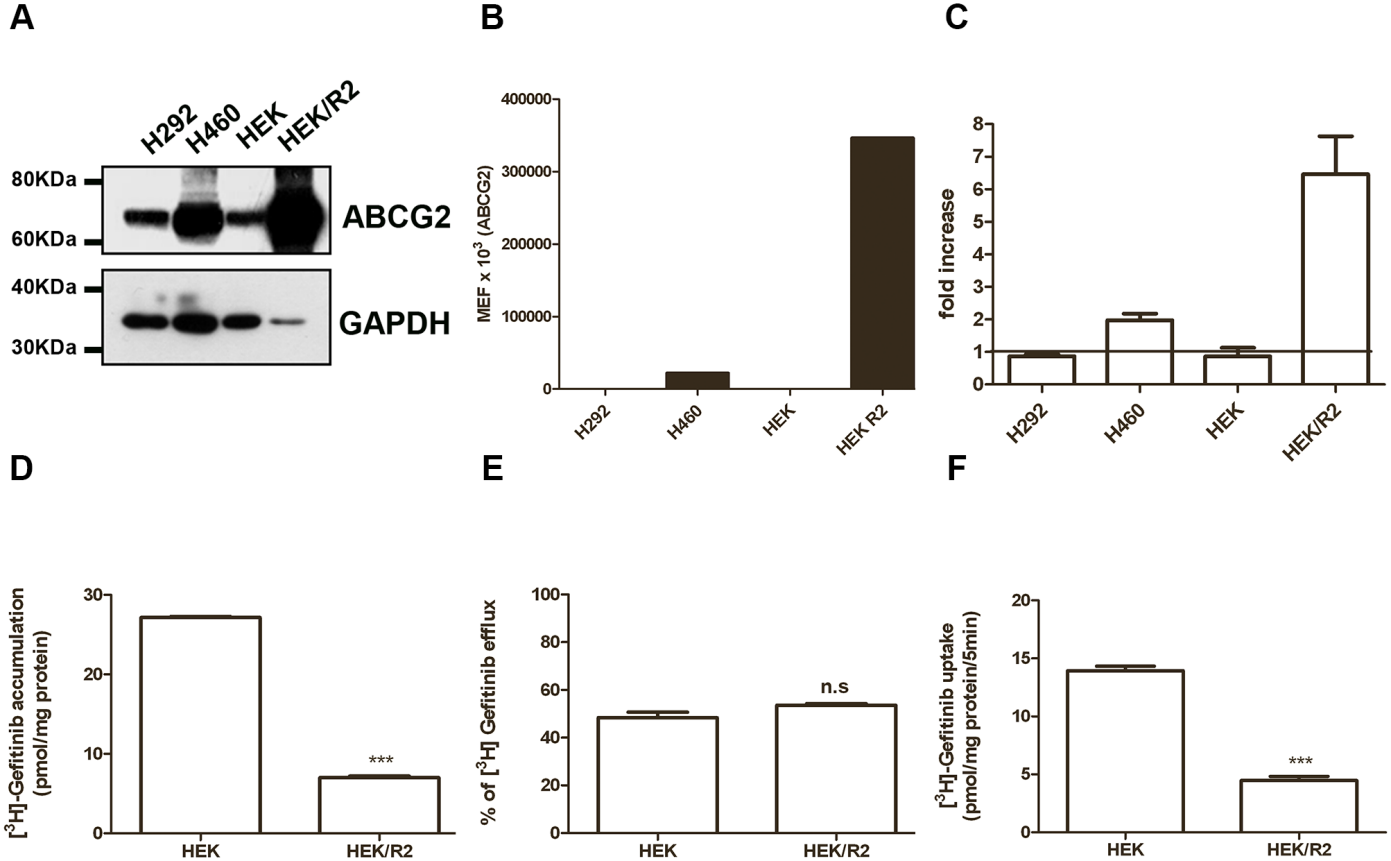
Effect of ABCG2 overexpression on gefitinib accumulation, efflux, and uptake. Characterization of HEK293/R2 overexpressing cells: (A) Cells were lysed and 50μg of proteins for H292, H460 and HEK293 or 10μg for HEK293/R2 cells loaded to assess ABCG2 protein expression by Western blot analysis. Different amounts of proteins were loaded to avoid the signal saturation in the sample from ABCG2 overexpressing cells. Data are from a representative experiment. Each experiment, repeated twice, yielded similar results. (B) ABCG2 protein levels on cell surface were quantified by flow-cytometry and expressed as molecular equivalent of fluorochrome (MEF) as described in the Methods Section. (C) Cells were loaded with 1 μM Hoechst 33342 in the presence or in the absence of 10 μM Fumitremorgin C. After 4 hours of incubation, Hoechst 33342 was removed and its fluorescence was determined by luminometer. Relative ABCG2 activity was defined as the ratio of Hoechst 33342 accumulation per μg of protein between Fumetrimorgin C treated cells and untreated cells and was expressed as fold increase. The mean values of three independent measurements (± SD) are shown. The cellular accumulation (D), efflux (E) and uptake (F) of 0.1 μM [^3^H]gefitinib was determined in overexpressing ABCG2 transporter HEK293/R2 cells and in the parental cells (HEK293), after 4 hours of gefitinib treatment. The percentage of efflux was calculated after 5 min of incubation with fresh medium, while initial velocity (5 min) of [^3^H] gefitinib uptake was measured after 4 hours of 0.1 μM gefitinib treatment (***P < 0.001).

The level of ABCG2 protein in HEK293 and HEK293/R2 was evaluated by Western blotting and by flow cytometry (Fig 5A and 5B) and was compared with that expressed in H460 and H292 cells. HEK293/R2 cells showed a significant higher level of ABCG2 total protein and a 15-fold increase in the plasma membrane level in respect to H460 cells. To assess the activity of ABCG2 protein, we measured the Hoechst 33342 dye accumulation in the absence or presence of the specific ABCG2 inhibitor, Fumitremorgin C (Fig 5C). After 4 hours of incubation with 1 μM Hoechst 33342 in the presence of 10 μM Fumitremorgin C, H460 cells lines showed 2-fold increase in the intracellular dye level compared with 7-fold increase in HEK293/R2.

The intracellular levels of radiolabeled gefitinib in HEK293 and HEK293/R2 cells were determined after 4 hours-exposure to [^3^H]gefitinib. In the parental HEK293 cells, [^3^H]gefitinib reached values up to 27 pmol/mg of protein (± 0.139), while in HEK293/R2 cells [^3^H]gefitinib accumulated up to 7 pmol/mg of protein (± 0.212) (Fig 5D).

This result was in agreement with data from literature demonstrating that in ABCG2 overexpressing cells gefitinib accumulated to a reduced level [20, 37, 38].

Then, we performed efflux studies loading parental HEK293 and HEK293/R2 cells with [^3^H]gefitinib for 4 hours and then removing gefitinib for 1 hour, by washing the cells with gefitinib-free normal growth medium. In accordance with the previous results, the percentage of efflux of the radiolabeled compound was similar in both the cell models (Fig 5E). Next, we investigated 5 min of [^3^H]gefitinib uptake in HEK-293/R2 and in parental cells, treated with gefitinib for 4 hours (Fig 5F). Interestingly, overexpression of ABCG2 strongly inhibited the [^3^H]gefitinib uptake in HEK293/R2 cells.

All together these results indicate that ABCG2 expression is able to affect [^3^H]gefitinib accumulation; however, despite the relevance of ABCG2 as efflux pump, we suggest that its overexpression is associated with a reduced gefitinib uptake mediated by a transporter that has not yet been characterized [28].

### Effect of ABCG2 overexpression on drug transporter expression level and uptake of different substrates

Based on the results shown in Fig 5 and since it has been reported that overexpression of efflux proteins may affect the activity of other influx transporters, by exerting either inhibition (Peptide transportes sysyem, PepT) [27] or activation (OCT-2) [39], we used a PCR array to compare mRNA levels of different drug transporters in extracts from HEK293 and HEK293/R2 cells. The fold change ±SD in the expression of 53 human drug transporters (mainly ABC and SLC genes) was reported in S1 Table. Transporters excluded from the table exhibited crossing points greater than the set threshold of 35. As expected, ABCG2 was found to be upregulated 65±16 fold in HEK292/R2 compared to HEK293 cells but the majority of transporters were down-regulated with fold decreases ranging from approximately -2 up to -20.

**Fig 6:**
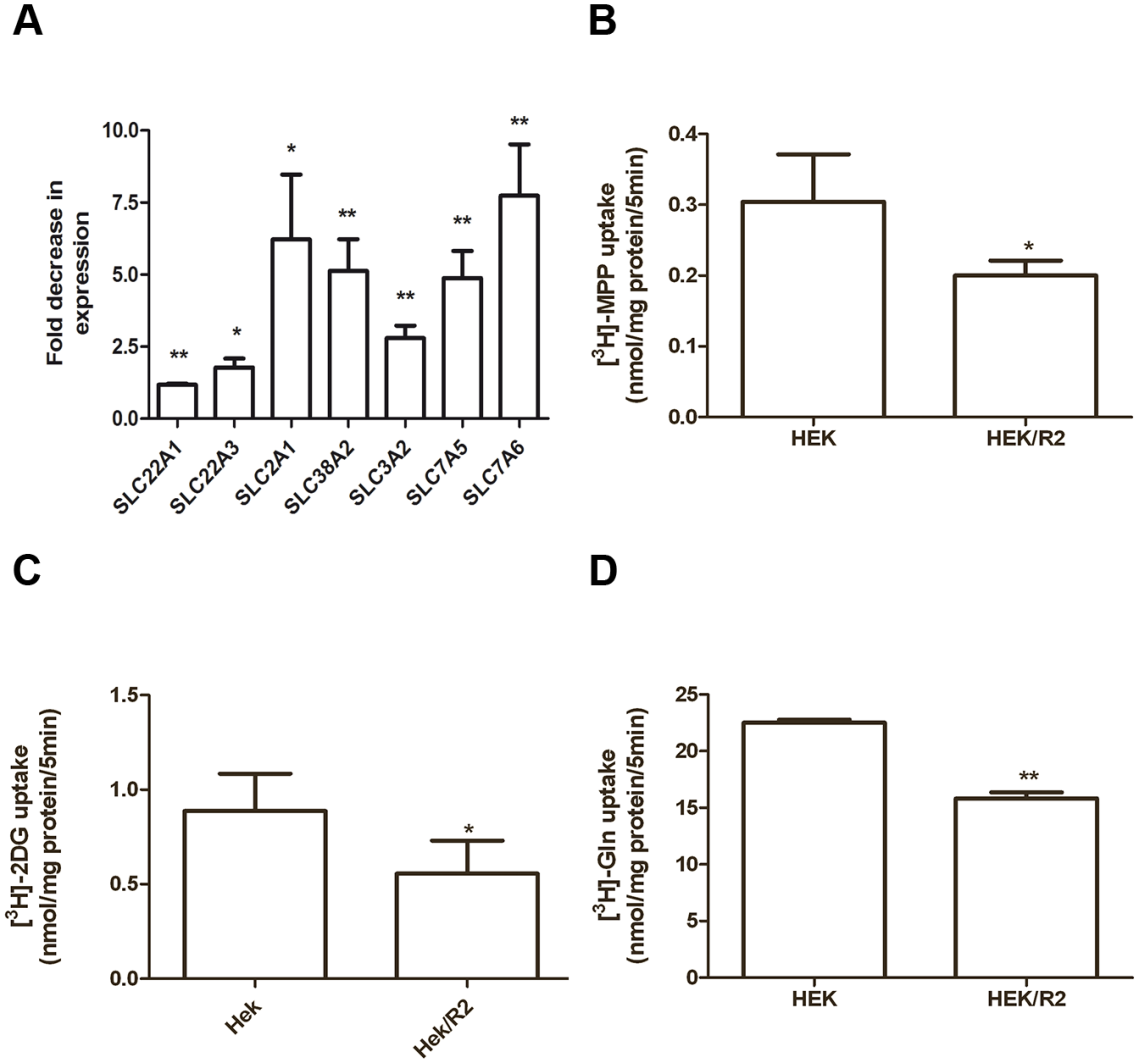
Effect of ABCG2 overexpression on the expression of SLC transporter genes and MPP, glucose and glutamine transport. Down-regulation of SLC transporter expression (SLC22A1, SLC22A3, SLC2A1, SLC38A2, SLC3A2, SLC7A5, and SLC7A6) in HEK293/R2 cells versus HEK293 was evaluated by PCR drug transporter array (A). Fold decrease expression was determined by the ΔΔC_T_ method normalized to five housekeeping genes. Values given are the means (± SD) of three independent experiments (*P<0.05; **P<0.01). Initial velocity (5 min) of [^3^H]MPP (B), [^3^H]2-deoxy-D-glucose (C) and [^3^H]Glutamine (D) uptake was determined and values given are the means (± SD) of three independent determinations (*P<0.05; **P<0.01).

We then correlated the variation in the expression of MPP transporters (SLC22A1 and SLC22A3, Solute carrier family 22), glucose transporter (SLC2A1, Facilitate glucose transporter) and glutamine transporters (SLC38A2, SLC3A2, SLC7A5, and SLC7A6) (Fig 6A) with the uptake of MPP, 2-deoxy-D-glucose and glutamine (Fig 6B, 6C and 6D).

Our results demonstrated that the down-regulation in the expression of the transporters in HEK293/R2 cells was associated with a decrease in the MPP, glucose, and glutamine uptake.

These findings suggest that changes in ABC and SLC transporter expression in cells overexpressing ABCG2 may contribute to differentially regulate the influx of nutrients and drugs, including gefitinib, into the cells.

## Discussion

The overexpression of the efflux transporter ABCG2 confers resistance to various chemotherapeutic drugs including new targeted molecules such as TKIs. On the other hand, TKIs can overcome resistance to chemotherapeutics by modulating ABC transporter activity [8, 9]. It has been reported that several TKIs (imatinib, nilotinib, dasatinib, lapatinib, sunitinib, erlotinib and gefitinib) are substrates or modulators of MDR1 and ABCG2, depending on their concentration and affinity for the transporters [8–14] Generally, extrusion has been shown to occur at low TKIs concentrations, while transporter inhibition is prevalent at higher concentrations [15].

Regarding the nature of the interaction between gefitinib and ABCG2 many discrepancies have emerged. Resistance to gefitinib has been attributed to the increased expression of ABCG2 [21, 40]. However, the demonstration that gefitinib is actively extruded from the cells derived mainly from indirect observations. For example, Chen et al [21] evaluated gefitinib efflux by collecting conditioned media from human epidermoid carcinoma A431 cell clones expressing different level of ABCG2, by exposing MDA-MB-468 cells to these media, and finally by evaluating the inhibition of EGFR Tyrosine1068 phosphorylation.

More direct approaches indicated that gefitinib is an inhibitor but not a substrate for ABCG2. *In vitro* gefitinib potently reversed resistance to SN-38 in Saos2 cell line engineered to overexpress functional ABCG2. However, overexpression of ABCG2 did not decrease accumulation nor increase the rate of efflux of [^14^C]gefitinib [22]. Moreover, equal expression of a Walker mutant, that is not a functional transporter in these cells, didn’t affect the uptake and efflux of [^14^C]gefitinib when compared to cells carrying functional ABCG2. Authors concluded that gefitinib inhibits ABCG2 but is not a substrate for ABCG2-mediated efflux.

In addition, by using plasma membrane vesicles derived from PC6/SN2-5H cells, gefitinib was found to inhibit the topotecan transport, without being transported into the vesicles [25]; in the same study, the analysis of kinetic parameters showed that gefitinib reversed ABCG2-mediated drug resistance by direct inhibition instead of competitive inhibition as an ABCG2 substrate. Moreover, Lemos et al. [41] demonstrated that high expression of ABCG2 in Caco2 cells was not associated with gefitinib resistance and that the sensitivity to gefitinib did not change in the absence or in the presence of the ABCG2 inhibitor Ko143.

In the present paper we analysed the interaction between ABCG2 and gefitinib in a panel of NSCLC cell lines, either sensitive or resistant to gefitinib. In particular, we studied whether gefitinib, in a range between 10 nM and 10 μM, inhibited the function of the transporter and whether ABCG2 silencing or overexpression modified the intracellular level of gefitinib as a result of an altered efflux of the drug.

In the first set of experiments we demonstrated that the expression of ABCG2 varied widely among the cell lines tested both as total amount of protein and as expression on the plasma membrane, without any correlation with sensitivity to gefitinib. In H460, SKMES-1 and A549 cells, in which ABCG2 was expressed at detectable level, we observed an increase in the intracellular accumulation of Hoechst 33342 in the presence of the specific ABCG2 inhibitor Fumitremorgin C, indicating that the transporter was functionally active. Then, we studied the effect of gefitinib on ABCG2 activity in H460 cells, demonstrating that gefitinib inhibited the efflux of Hoechst 33342 in a dose-dependent manner starting from 100 nM, without any variation in the plasma membrane level of ABCG2. This result suggests that gefitinib can directly interact with ABCG2, although it does not clarify whether the mechanism of this interaction involves a competition with the substrate or a direct inhibition of the carrier.

Some authors reported that gefitinib is transported by ABCG2 at low doses (nanomolar), whereas it is an inhibitor at higher concentrations (micromolar range) [20, 38].

To evaluate whether gefitinib can act as an ABCG2 substrate in our experimental system, we incubated H460 cells with very low concentrations of the drug (10nM in Fig 3A and 100nM in Fig 3C–3H), and analysed the effects of different ABCG2 inhibitors on its intracellular accumulation.

As expected, Fumitremorgin C increased the level of Hoechst 33342; however, neither Fumitremorgin C and Ko143 (ABCG2 inhibitors) nor PSC833 (MDR1 inhibitor) increased the intracellular level of gefitinib, evaluated by both radioactivity dosage and LC-MS/MS analysis. Correspondingly, the percentage of efflux was unchanged in the absence or presence of the inhibitors, a result also confirmed in other NSCLC cell lines showing different ABCG2 expression. This finding strongly suggests that gefitinib is not a substrate of ABCG2 even at low concentrations. Anyway, it is worth of note that gefitinib has a very high distribution volume (1400 litres) with an extensive distribution in tissues, including lung and tumours, where it can reach concentrations 10 fold higher than in plasma [42, 43]. In addition, our previous results indicated that intracellular gefitinib concentrations are more than 200 times higher than outside the cells [28, 29]. Therefore, the effective clinical concentration of gefitinib in the local tumour environment is presumably in the range of micromolar, suggesting that this drug is more likely to act as an ABCG2 inhibitor in vivo. This is an important aspect that should be considered in the interpretation of the in vitro results.

The first set of experiments in this study was performed in NSCLC cells expressing endogenous levels of ABCG2. Since previous data on ABCG2-drug interactions mostly derived from studies on ABCG2 overexpressing cell models, we evaluated whether a forced modulation of ABCG2 expression, by either silencing or overexpressing the protein, may affect gefitinib efflux and accumulation. To this end we silenced ABCG2 in H460 cells and used HEK293/R2 as a cell model overexpressing ABCG2. We actually found that after silencing or overexpressing ABCG2 the intracellular content of gefitinib was significantly increased and decreased, respectively. These changes in gefitinib accumulation, however, were not due to an altered efflux of the drug, being instead associated with a modulation of the uptake process. In this context, it is worth of note that blasts from acute lymphoblastic leukemia with high ABCG2 expression showed a very high resistance to cytarabine despite the fact that cytarabine is not an ABCG2 substrate [44].

The intracellular concentration of a compound is modulated by the activity of both ABC transporters serving as efflux pumps, and the members of the solute carrier family acting as uptake transporters. We have previously demonstrated that the uptake of gefitinib is essentially an active, temperature-dependent, sodium- and potential-independent process [28]. Here we show that the uptake of gefitinib measured as initial velocity (5 min) was enhanced in ABCG2 silenced cells and strongly reduced in ABCG2 overexpressing cells, suggesting that ABCG2 expression may modulate the activity and/or the level of a transporter involved in the entrance of gefitinib into the cells. To confirm the hypothesis that overexpression of ABCG2 efflux transporter may affect the function of other endogenous influx transporters, we performed a PCR array to assess changes in the expression of 84 transporter genes. As previously reported for the ^+^H/peptide transporter PepT2 (SCL1SA2) [27], many transporters, mainly members of ABC and SLC family, appeared to be down-regulated as a result of ABCG2 overexpression and, in accordance, overexpression of ABCG2 strongly reduced the uptake, as initial velocity rate, of MPP, glucose and glutamine.

The molecular mechanism underlying the interaction between the overexpressed ABCG2 transporter and other influx transporters has not been elucidated yet. One possible explanation is that ABCG2 gene, when abundantly expressed, may compete with other endogenous genes at transcriptional level and our results based on RT^2^ profiler PCR array of human drug transporters seem to confirm this hypothesis.

In addition, a competition at translational or post-translational level may also occur due to a crosstalk among the different membrane transporters. Further detailed studies are required to examine the functional relevance of such interactions.

Considering that normal and cancer stem cells are frequently identified based on their low accumulation of Hoechst 33342 dye due to ABCG2 overexpression [45], we can speculate that, in addition to resistance to chemotherapeutics drugs, ABCG2 may also control other important stem cell properties by modulating the influx and accumulation of different substrates including nutrients, with a selective advantage against neighbouring cells.

## Supporting Information

S1 Fig Effect of ABCG2 silencing with a single siRNA on ABCG2 expression, activity, [^3^H]gefitinib accumulation and uptake. (DOCX)

S1 Table Fold changes in transporter expression in HEK293/R2 overexpressing ABCG2 cells compared to HEK293 parental cells using RT2 profiler PCR array. (DOCX)
