# Supplementary material for: Effect of ABCG2/BCRP Expression on Efflux and Uptake of Gefitinib in NSCLC Cell Lines
Source: PLoS One. 2015 Nov 4;10(11):e0141795. doi: 10.1371/journal.pone.0141795 (PMC4633241; doi:10.1371/journal.pone.0141795)
Supplement: S1 Fig — (DOCX) [file pone.0141795.s001.docx]

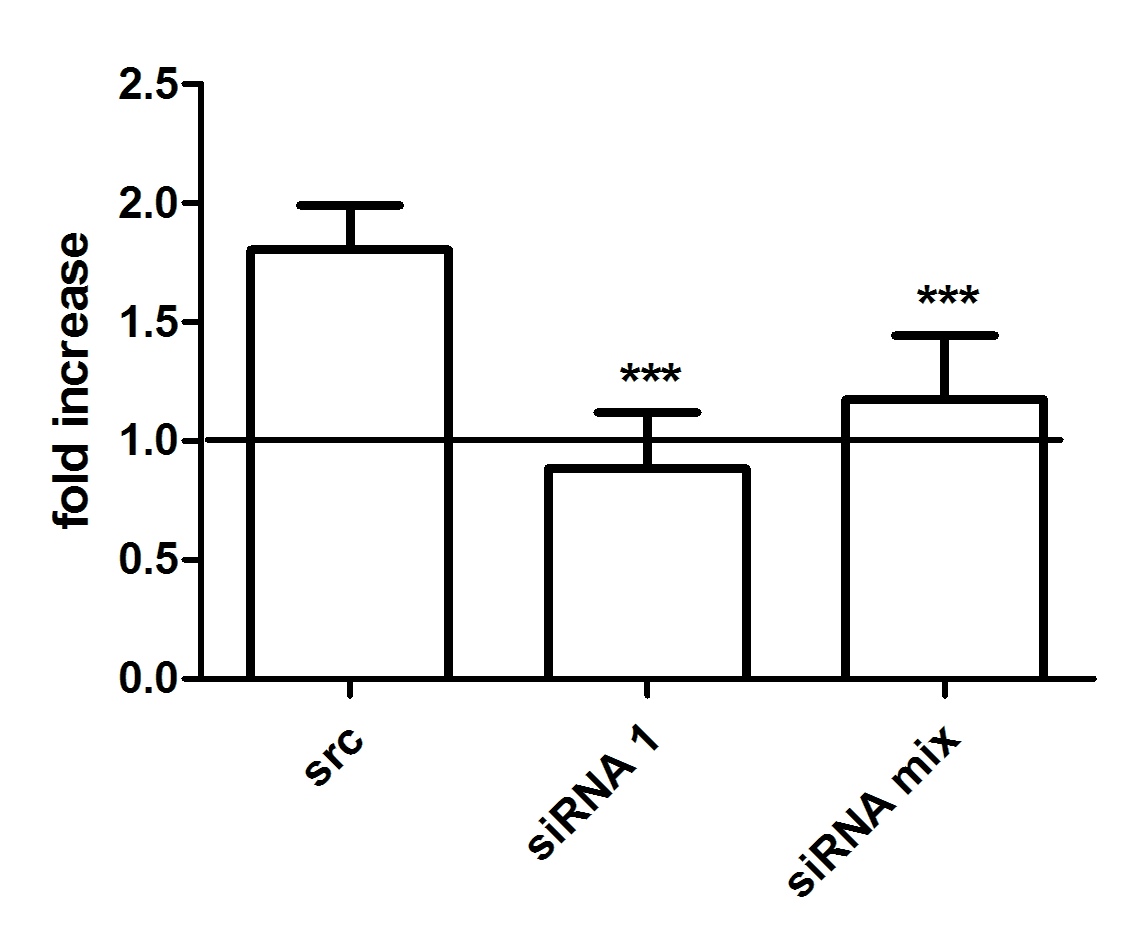

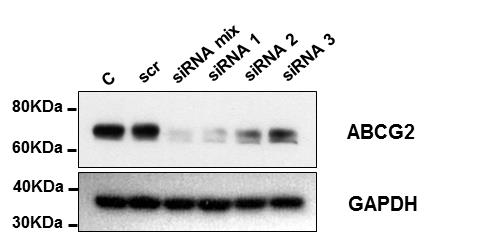


**B**

**A**

**D**

**C**


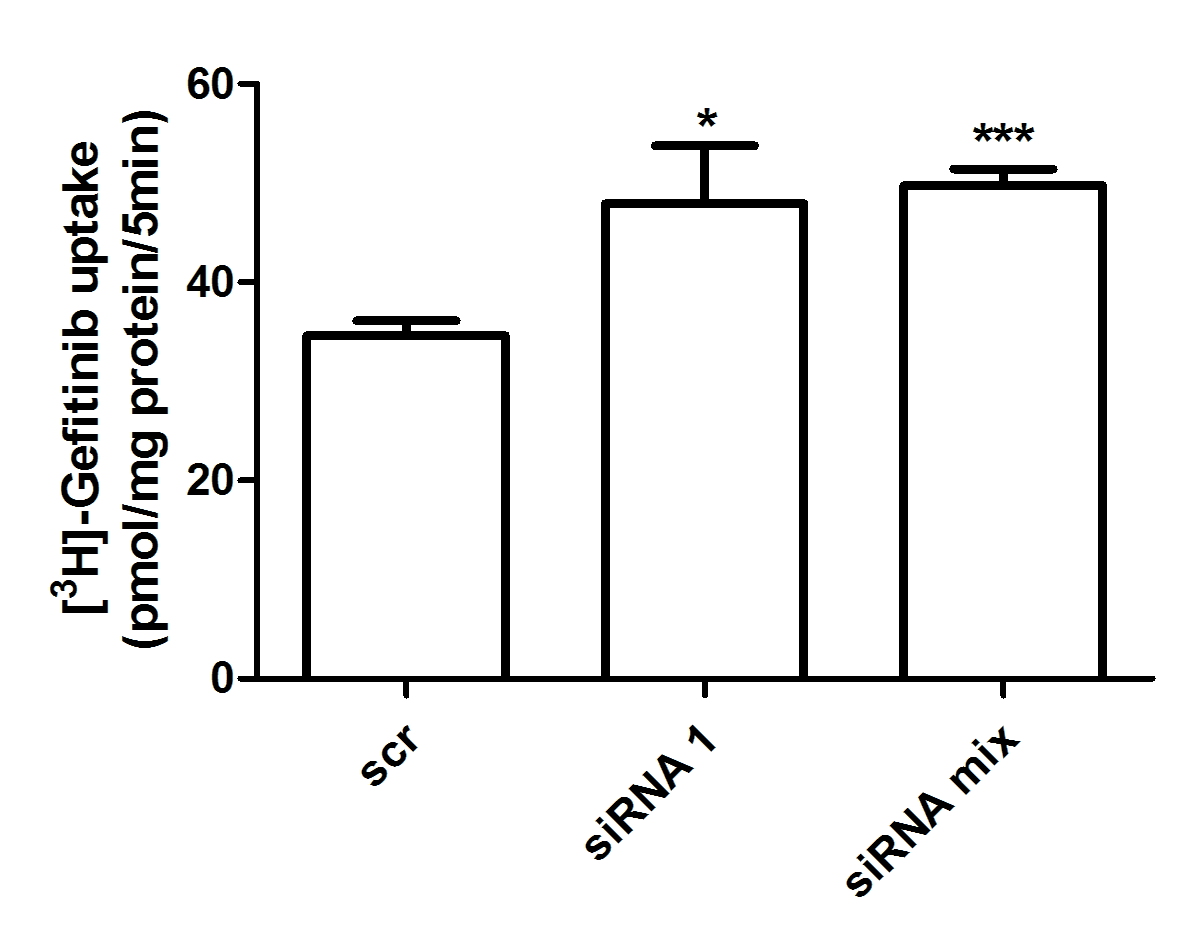

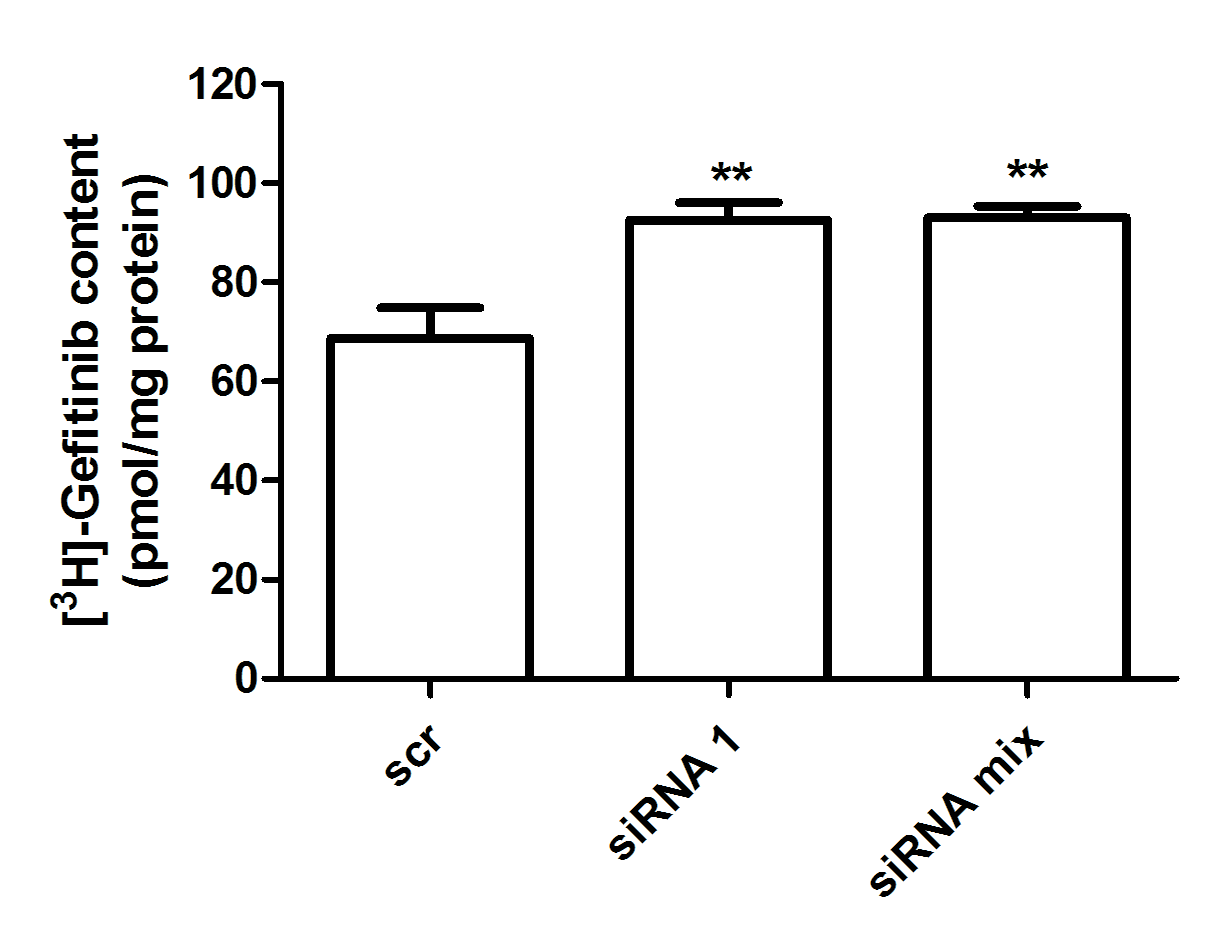


**S1 Fig. Effect of ABCG2 silencing with a single siRNA on ABCG2 expression, activity, [^3^H]gefitinib accumulation and uptake.** H460 cells were transfected with siRNA mix [1:1:1 mixture of ^#^HSS114013 (ABCG2 siRNA1), ^#^HSS114014 (siRNA2) and ^#^HSS114015 (siRNA3)] or with single siRNAs or control siRNA (scr) for 48 hours and then analyzed for ABCG2 expression by Western blotting (A) or ABCG2 activity (B) as described in Material and Methods. Data are expressed as mean (± SD) of three different experiments (***P < 0.001). In silenced cells (siRNA1 or siRNAmix), radiolabeled gefitinib accumulation (C) and initial velocity (5 min) of [^3^H] gefitinib uptake (D) were measured. Each bar represents the mean (± SD) of four independent determinations (*P < 0.05; ** P < 0.01; ***P < 0.001).
