# Supplementary material for: Effect of ABCG2/BCRP Expression on Efflux and Uptake of Gefitinib in NSCLC Cell Lines
Source: PLoS One. 2015 Nov 4;10(11):e0141795. doi: 10.1371/journal.pone.0141795 (PMC4633241; doi:10.1371/journal.pone.0141795)
Supplement: S1 Table — (DOCX) [file pone.0141795.s002.docx]

| Gene | Gene bank number | Protein | Fold change |
| --- | --- | --- | --- |
| ABCA12 | NM_173076 | ATP-binding cassette, sub-family A (ABC1), member 12 | -4.04 ± 0.67 |
| ABCA2 | NM_001606 | ATP-binding cassette, sub-family A (ABC1), member 2 | -8.13 ± 1.68 |
| ABCA5 | NM_018672 | ATP-binding cassette, sub-family A (ABC1), member 5 | -3.99 ± 0.13 |
| ABCB1 | NM_000927 | ATP-binding cassette, sub-family B (MDR/TAP), member 1 | -4.61 ± 1.60 |
| ABCB6 | NM_005689 | ATP-binding cassette, sub-family B (MDR/TAP), member 6 | -2.19 ± 0.08 |
| ABCC1 | NM_004996 | ATP-binding cassette, sub-family C (CFTR/MRP), member 1 | -7.71 ± 3.02 |
| ABCC10 | NM_033450 | ATP-binding cassette, sub-family C (CFTR/MRP), member 10 | -12.50 ± 6.77 |
| ABCC2 | NM_000392 | ATP-binding cassette, sub-family C (CFTR/MRP), member 2 | -2.01 ± 0.25 |
| ABCC4 | NM_005845 | ATP-binding cassette, sub-family C (CFTR/MRP), member 4 | -3.84 ± 0.34 |
| ABCC5 | NM_005688 | ATP-binding cassette, sub-family C (CFTR/MRP), member 5 | -10.48 ± 0.26 |
| ABCD1 | NM_000033 | ATP-binding cassette, sub-family D (ALD), member 1 | -9.31 ± 3.51 |
| ABCD3 | NM_002858 | ATP-binding cassette, sub-family D (ALD), member 3 | -7.20 ± 1.48 |
| ABCD4 | NM_005050 | ATP-binding cassette, sub-family D (ALD), member 4 | -3.13 ± 0.06 |
| ABCF1 | NM_001090 | ATP-binding cassette, sub-family F (GCN20), member 1 | -4.98 ± 0.73 |
| ABCG2 | NM_004827 | ATP-binding cassette, sub-family G (WHITE), member 2 | 65.39 ± 16.43 |
| AQP1 | NM_198098 | Aquaporin 1 (Colton blood group) | -1.42 ± 0.57 |
| ATP6V0C | NM_001694 | ATPase, H+ transporting, lysosomal 16kDa, V0 subunit c | -2.06 ± 0.24 |
| ATP7A | NM_000052 | ATPase, Cu++ transporting, alpha polypeptide | -9.81 ± 0.97 |
| ATP7B | NM_000053 | ATPase, Cu++ transporting, beta polypeptide | -8.98 ± 3.49 |
| MVP | NM_017458 | Major vault protein | -2.27 ± 0.20 |
| SLC15A1 | NM_005073 | Solute carrier family 15 (oligopeptide transporter), member 1 | -2.74 ± 0.12 |
| SLC15A2 | NM_021082 | Solute carrier family 15 (H+/peptide transporter), member 2 | -5.14 ± 0.40 |
| SLC16A1 | NM_003051 | Solute carrier family 16, member 1 (monocarboxylic acid transporter 1) | -5.76 ± 1.25 |
| SLC16A2 | NM_006517 | Solute carrier family 16, member 2 (monocarboxylic acid transporter 8) | -13.52 ± 2.28 |
| SLC19A1 | NM_194255 | Solute carrier family 19 (folate transporter), member 1 | -2.6 ± 0.22 |
| SLC19A2 | NM_006996 | Solute carrier family 19 (thiamine transporter), member 2 | -7.12 ± 0.10 |
| SLC22A1 | NM_003057 | Solute carrier family 22 (organic cation transporter), member 1 | -1.18 ± 0.04 |
| SLC22A3 | NM_021977 | Solute carrier family 22 (extraneuronal monoamine transporter), member 3 | -1.78 ± 0.31 |
| SLC22A7 | NM_006672 | Solute carrier family 22 (organic anion transporter), member 7 | -1.28 ± 0.17 |
| SLC25A13 | NM_014251 | Solute carrier family 25, member 13 (citrin) | -8.27 ± 2.09 |
| SLC28A1 | NM_004213 | Solute carrier family 28 (sodium-coupled nucleoside transporter), member 1 | -1.32 ± 0.27 |
| SLC29A1 | NM_004955 | Solute carrier family 29 (nucleoside transporters), member 1 | -4.54 ± 0.49 |
| SLC29A2 | NM_001532 | Solute carrier family 29 (nucleoside transporters), member 2 | -4.50 ± 0.08 |
| SLC2A1 | NM_006516 | Solute carrier family 2 (facilitated glucose transporter), member 1 | -6.22 ± 2.25 |
| SLC2A3 | NM_006931 | Solute carrier family 2 (facilitated glucose transporter), member 3 | -20.55 ± 6.42 |
| SLC31A1 | NM_001859 | Solute carrier family 31 (copper transporters), member 1 | -7.35 ± 2.59 |
| SLC38A2 | NM_018976 | Solute carrier family 38, member 2 | -5.13 ± 1.10 |
| SLC3A1 | NM_000341 | Solute carrier family 3 (cystine, dibasic and neutral amino acid transporters, activator of cystine, dibasic and neutral amino acid transport), member 1 | -6.72 ± 1.71 |
| SLC3A2 | NM_002394 | Solute carrier family 3 (activators of dibasic and neutral amino acid transport), member 2 | -2.80 ± 0.43 |
| SLC5A4 | NM_014227 | Solute carrier family 5 (low affinity glucose cotransporter), member 4 | -3.48 ± 0.24 |
| SLC7A11 | NM_014331 | Solute carrier family 7 (anionic amino acid transporter light chain, xc- system), member 11 | -9.65 ± 3.10 |
| SLC7A5 | NM_003486 | Solute carrier family 7 (amino acid transporter light chain, L system), member 5 | -4.88 ± 0.94 |
| SLC7A6 | NM_003983 | Solute carrier family 7 (amino acid transporter light chain, y+L system), member 6 | -7.74 ± 1.78 |
| SLC7A7 | NM_003982 | Solute carrier family 7 (amino acid transporter light chain, y+L system), member 7 | -4.78 ± 0.53 |
| SLC7A8 | NM_182728 | Solute carrier family 7 (amino acid transporter light chain, L system), member 8 | -1.46 ± 0.28 |
| SLC7A9 | NM_014270 | Solute carrier family 7 (glycoprotein-associated amino acid transporter light chain, bo,+ system), member 9 | -2.33 ± 1.15 |
| SLCO2A1 | NM_005630 | Solute carrier organic anion transporter family, member 2A1 | -6.38 ± 0.67 |
| SLCO3A1 | NM_013272 | Solute carrier organic anion transporter family, member 3A1 | -5.32 ± 1.77 |
| SLCO4A1 | NM_016354 | Solute carrier organic anion transporter family, member 4A1 | -2.65 ± 0.49 |
| TAP1 | NM_000593 | Transporter 1, ATP-binding cassette, sub-family B (MDR/TAP) | -3.62 ± 0.42 |
| TAP2 | NM_000544 | Transporter 2, ATP-binding cassette, sub-family B (MDR/TAP) | -4.50 ± 0.42 |
| VDAC1 | NM_003374 | Voltage-dependent anion channel 1 | -2.98 ± 0.13 |
| VDAC2 | NM_003375 | Voltage-dependent anion channel 2 | -1.80 ± 0.16 |

**S1 Table. Fold changes in transporter expression in HEK293/R2 overespressing ABCG2 cells compared to HEK293 parental cells using RT^2^ profiler PCR array.**
